# Angiographic Patency of Coronary Artery Bypass Conduits: An Updated Network Meta-Analysis of Randomized Trials

**DOI:** 10.21470/1678-9741-2022-0142

**Published:** 2022

**Authors:** Mimi X. Deng, Hillary Lia, Grace Lee, Mohamed Rahouma, Antonino Di Franco, Michelle Demetres, Gianni D. Angelini, Mario Gaudino, Stephen E. Fremes

**Affiliations:** 1 Division of Cardiac Surgery, Schulich Heart Centre, Department of Surgery, Sunnybrook Health Sciences Centre, University of Toronto, Toronto, Ontario, Canada; 2 Temerty School of Medicine, University of Toronto, Toronto, Ontario, Canada; 3 Department of Cardiothoracic Surgery, Weill Cornell Medicine, New York, New York, United States of America; 4 Samuel J. Wood Library & C.V. Starr Biomedical Information Center, Weill Cornell Medicine, New York, New York, United States of America; 5 Bristol Heart Institute, University of Bristol, Bristol, United Kingdom

**Keywords:** Coronary Artery Bypass, Coronary Artery Bypass Grafting, Angiography, Graft Patency, Coronary Artery Disease.

## Abstract

**Introduction:**

The second best conduit for coronary artery bypass grafting is uncertain. The objective of this study is to determine the second best conduit according to graft patency results from randomized controlled trials using a network meta-analysis.

**Methods:**

A systematic literature search was conducted for randomized controlled trials comparing the angiographic patency rate of the no-touch saphenous vein (NT-SV), the radial artery (RA), the right internal thoracic artery (RITA), and the gastroepiploic artery (GEA) in reference to the conventionally harvested saphenous vein (CON-SV). The primary outcome was graft occlusion, and the secondary outcome was all-cause mortality.

**Results:**

A total of 859 studies were retrieved, of which 18 were included. A total of 6,543 patients and 8,272 grafts were analyzed. The weighted mean angiographic follow-up time was 3.5 years. Compared with CON-SV, RA (incidence rate ratio [IRR] 0.56; 95% confidence interval [CI], 0.43-0.74) and NT-SV (IRR 0.56; 95% CI, 0.44-0.70) demonstrated lower graft occlusion. NT-SV and RA were ranked as the best conduits (rank score for NT-SV 0.88 vs. 0.87 for RA, 0.29 for GEA, 0.27 for CON-SV, and 0.20 for RITA). There was no significant difference in late mortality between different conduit types.

**Conclusion:**

RA and NT-SV are associated with significantly lower graft occlusion rates and are comparably ranked as the best conduit for patency.

**Table:** 

Abbreviations, Acronyms & Symbols			
ART	= Arterial Revascularization Trial	NR	= Not reported
BITA	= Bilateral internal thoracic artery	NT-SV	= No-touch saphenous vein
BMI	= Body mass index	OR	= Odds ratio
CABG	= Coronary artery bypass grafting	RA	= Radial artery
CAD	= Coronary artery disease	RADIAL	= Radial Artery Database International Alliance
CI	= Confidence interval	RAPCO	= Radial Artery Patency and Clinical Outcomes
CON-SV	= Conventionally harvested saphenous vein	RAPS	= Radial Artery Patency Study
CTA	= Computed tomography angiography	RCA	= Right coronary artery
EuroSCORE	= European System for Cardiac Operative Risk Evaluation	RCTs	= Randomized controlled trials
FEV1	= Forced expiratory volume in 1 second	RIMA	= Right internal mammary artery
GEA	= Gastroepiploic artery	RITA	= Right internal thoracic artery
IRR	= Incidence rate ratio	RSVP	= Radial Artery Versus Saphenous Vein Patency
ISR	= In-stent restenosis	SAVE-RITA	= Saphenous Vein versus Right Internal Thoracic Artery
ITA	= Internal thoracic artery	SD	= Standard deviation
IVUS	= Intravascular ultrasound	seTE	= Standard error of treatment estimate
LAD	= Left anterior descending	SV	= Saphenous vein
LITA	= Left internal thoracic artery	SVG	= Saphenous vein graft
LVEF	= Left ventricular ejection fraction	TE	= Estimate of treatment effect
NMA	= Network meta-analysis	TIMI	= Thrombolysis in myocardial infarction

## INTRODUCTION

The long-term benefit of coronary artery bypass grafting (CABG) is dependent on durable patency of the conduits used. The left internal thoracic artery (LITA) to left anterior descending (LAD) bypass is universally accepted as the gold-standard that confers the greatest survival benefit. Between a selection of arterial grafts and the saphenous vein, the second conduit of choice remains controversial^[^1^]^.

Compared to the saphenous vein grafts, arterial grafts are advocated for long-term patency and resistance to progressive graft atherosclerosis^[^2^]^. However, minimal handling of the saphenous vein during harvesting has provided vein graft patency rates that are on par with their arterial counterparts^[^3^]^. A comprehensive network meta-analysis (NMA) of graft patency in randomized controlled trials (RCTs) was previously completed by our group^[^4^]^. The key findings were that the radial artery (RA) and no-touch saphenous vein (NT-SV) grafts were associated with significantly lower graft occlusion rates compared with the conventionally harvested saphenous vein (CON-SV), with RA demonstrating the best patency^[^4^]^. The systematic review of this study was completed in 2019. Since then, additional RCTs with pairwise comparisons of two or more conduit types have been published (including one very large study comparing CON-SV and NT-SV)^[^3^]^, and previous studies have been updated with long-term results^[^2^,^5^,^6^]^. We have therefore updated the previously published NMA of the RCTs comparing graft patency of all conduit options in CABG, in an effort to provide high-level evidence to guide graft selection.

## METHODS

No human subjects were involved; therefore, ethical approval of this analysis was not required. The data that support the findings of this study are available from the corresponding author upon request.

### Search Strategy

For the previous NMA^[^4^]^, a medical librarian (M.D.) had performed a comprehensive literature search, on November 11, 2019, of RCTs that compared CON-SV, NT-SV, RA, the right internal thoracic artery (RITA), or the gastroepiploic artery (GEA). For this NMA, the same librarian performed an updated search on December 22, 2021 in the following databases: Ovid® MEDLINE®, Ovid® EMBASE®, and the Cochrane Library. The search strategy included the terms “radial artery”, “internal mammary artery”, “internal thoracic artery”, “gastroepiploic artery”, and “saphenous vein”. The full search strategy is available in Table S1. This review was registered with the PROSPERO register of systematic reviews (CRD42022303553).

**Table S1:** Search Strategy.

Ovid® MEDLINE® (ALL - 1946 to December 22, 2021).
Searched on 12/22/2021. Limited to English language RCTs.
Line# | Search
Radial Artery/
(radial arter^*^ or arteria radialis or radialis artery).tw.
Saphenous Vein/
(Saphenous or SVG or saphena vein or saphenous venos system or vena saphena).tw.
Internal Mammary-Coronary Artery Anastomosis/
(Right Internal Mammary Artery or RIMA or Coronary Internal Mammary Artery or arteria mammaria interna or arteria thoracica interna or right internal thoracic artery or mammary internal artery).tw.
(cardiac muscle revascularisation or cardiac muscle revascularization or coronary revascularisation or coronary revascularization or heart muscle revascularisation or heart myocardium revascularisation or heart revascularisation or heart revascularization or internal mammary arterial anastomosis or internal mammary arterial implantation or internal mammary artery anastomosis or internal mammary artery graft or internal mammary artery implant or internal mammary artery implantation or internal mammary-coronary artery anastomosis or myocardial revascularisation or myocardial revascularization or myocardium revascularisation or myocardium revascularization or transmyocardial laser revascularisation or transmyocardial laser revascularization or vineberg operation).tw.
Gastroepiploic Artery/
(gastroepiploic artery or gastroepiploic arteries or gastroepiploic blood vessel or arteria gastroepiploica).tw.
or/1-9
"randomized controlled trial".pt.
(randomized controlled trial or randomised controlled trial or randomized trial or randomised trial or single blind^*^ or double blind^*^ or triple blind^*^).ti,ab.
11or12
(animals not humans).sh.
(comment or editorial or meta-analysis or practice-guideline or review or letter).pt. or meta- analysis.ti.
(random sampl^*^ or random digit^*^ or random effect^*^ or random survey or random regression).ti,ab.not "randomized controlled trial".pt.
13not(14or15or16)
10 and 17
limit 18 to english language
RCTs=randomized controlled trials; RIMA=right internal mammary artery; SVG=saphenous vein graft

### Study Selection and Quality Assessment

Searches across the aforementioned databases retrieved 859 studies. After citations were de-duplicated, two independent reviewers (M.X.D and H.L.) screened a total of 577 references. Discrepancies were resolved by consensus and opinion of a third author (S.E.F.). Titles and abstracts were reviewed against predefined inclusion and exclusion criteria. Articles were appraised for eligibility if they were written in English and were RCTs randomized by conduit type, comparing angiographic patency for at least two of the five conduits (RA, RITA, CON-SV, NT-SV, and GEA) in patients undergoing CABG. Animal studies, case reports, conference presentations, editorials, expert opinions, observational studies, literature review, abstract only publications, and studies not defining or reporting the outcomes of interest were excluded. Two references that were previously acknowledged in the original NMA were removed to avoid duplication.

**Fig. S1:**
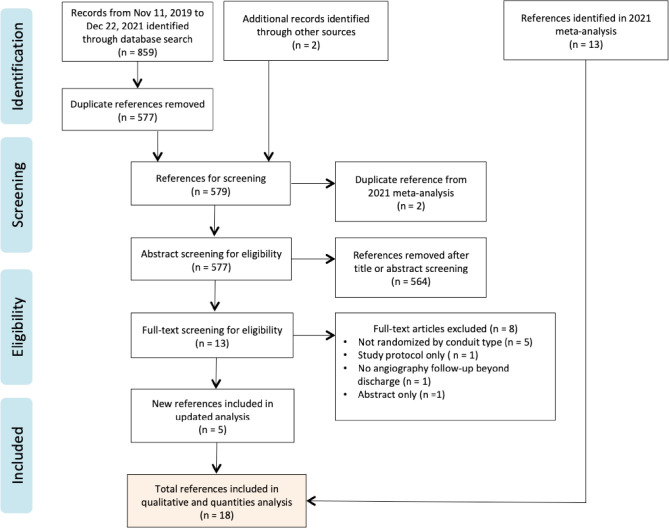
Preferred Reporting Items for Systematic Reviews and Meta-Analyses flow diagram.

Eligible abstracts proceeded to full-text review. The full flow diagram outlining the study selection process is shown in Figure S1. For overlapping studies involving the same study cohort with serial assessments over time, the study with the longest angiographic follow-up was included. The 13 studies reported in the original NMA were included in this updated review. The following variables were collected: study demographics (sample size, publication year, institution, country, and inclusion and exclusion criteria), patient demographics (age, sex, and comorbidities), procedure-related variables (number of grafts, distal anastomosis to the left circumflex artery, proximal anastomosis to the ascending aorta, and use of off-pump CABG), and angiographic-related variables (definition of graft occlusion, imaging modality, completeness of angiographic follow-up, and severity of the target vessel stenosis). The quality of the included trials was examined by the Cochrane Collaboration’s tool for assessing risk of bias^[^7^]^.

The primary outcome was graft occlusion at the protocol-defined angiographic follow-up. The secondary outcome was all-cause mortality.

### Statistical Analysis

The incidence rate with underlying Poisson process was used to account for different follow-up times among the studies, with the total number of events observed within a treatment group calculated out of the total person-time follow-up for that treatment group. Pooled crude graft patency results of the different graft types were performed using a random effects model and the generic inverse variance method. Random effects NMA using a frequentist approach was performed using the generic inverse variance method with CON-SV as reference. Pooled graft patency and late mortality were summarized as forest plots and league tables. Rank scores with probability ranks of different treatment groups were calculated for the primary outcome. Ranks closer to 1 indicate the probability that the treatment group leads to the greatest reduction in graft occlusion. Net graphs were constructed summarizing the numbers of direct comparisons of the included trials. Leave-one-out analysis for graft occlusion was done to assess for validity of the main analysis.

Subgroup analyses were performed for studies with target vessel stenosis ≥ 70% and studies that exclusively used computed tomography angiography (CTA) for postoperative graft assessment during follow-up.

The Cochran’s Q statistic was used to assess inconsistency using the decomposition approach. Inconsistencies were assessed based on separate indirect from direct evidence (or SIDE) using back-calculation method and decomposition of within-designs Q statistic. Net heat plot was used to evaluate for inconsistency in the network model. Heterogeneity was reported as low (I^^2^^ = 0-25%), moderate (I^^2^^ = 26-50%), or high (I^^2^^ > 50%).

Pairwise comparisons were also performed to assess the consistency of the network findings. Meta-regression was performed on the pairwise comparisons to explore the effect on the primary outcome of age, sex, hypertension, diabetes mellitus, dyslipidemia, target vessel stenosis, duration of follow-up, completeness of angiographic follow-up, percentage of proximal anastomoses on the ascending aorta, percentage of grafts to the circumflex coronary system, and use of off-pump CABG.

For hypothesis testing purposes, we built 95% confidence intervals (CI) without multiplicity adjustment. All statistical analyses were performed using the “meta” and “netmeta” packages of R (version 4.1.2, R Project for Statistical Computing using R Studio 2021.09.2).

## RESULTS

After removal of duplicates, a total of 577 studies were retrieved from the literature search. Two additional studies not identified in the initial search were included after professional consultation (S.E.F.)^[^3^,^6^]^. Of the 579 studies, 13 abstracts proceeded to full-text screen. Ultimately, five additional RCTs were included in the final analysis^[^3^,^6^,^8^-^10^]^. Together with the 13 RCTs from the original meta-analysis^[^2^,^5^,^11^-^21^]^, a total of 18 studies were included in this review (Table 1). The detailed inclusion and exclusion criteria of the individual trials are summarized in Table S2. Three trials were multicenter (two in Canada, one in the United States of America), two originated from Italy, two from Sweden, two from Korea, two from China, two from the United Kingdom, and one each from Belgium, Australia, Norway, Egypt, and Brazil. Two trials used within-patient randomization^[^12^,^14^]^. Both the RITA *vs.* RA (RAPCO-RITA) and the CON-SV *vs.* RA (RAPCO-SV) arms of the Radial Artery Patency and Clinical Outcomes (RAPCO) study were included^[^2^]^. In the 2005 trial by Gaudino et al.^[^15^]^, results of graft randomization in the study cohort of patients with coronary in-stent restenosis and the control cohort of patent stents were included. In the 2021 parallel group by Angelini et al.^[^8^]^, a factorial trial involving four treatment groups, only two of the groups were included - conventional harvest/high-pressure test and pedicled harvest/low-pressure test, representing CON-SV and NT-SV, respectively.

**Table 1:** Characteristics of included randomized trials.

Author, year	Institution	Country	Study Period	Number of Patients
Angelini, 2021^[8]^	Bristol Heart Institute and University of Bristol	United Kingdom	2009-2013	50
Buxton, 2020 (RAPCO)^[2]^	Austin Hospital and University of Melbourne	Australia	1996-2005	619
Collins, 2008 (RSVP)^[11]^	Royal Brompton Hospital	United Kingdom	1998-2000	142
Deb, 2012 (RAPS)^[12]^	Multicenter	Canada	1996-2001	510
Deb, 2019 (SUPERIOR SVG)^[13]^	Multicenter	Canada	2011-2013	250
Dreifaldt, 2019^[14]^	Department of Cardiovascular Surgery, University Hospital	Sweden	2004-2009	216
Gaudino, 2005^[15]^	Catholic University, Rome	Italy	1994-1997	120
Glineur, 2011^[16]^	Cliniques Universitaire St Luc.	Belgium	2003-2006	210
Goldman, 2011^[17]^	Multicenter	United States of America	2003-2009	757
Hou, 2021^[9]^	Beijing Anzhen Hospital	China	2018-2019	100
Kim, 2021 (SAVE-RITA)^[6]^	Seoul National University Hospital	South Korea	2008-2011	224
Muneretto, 2004^[18]^	University of Brescia Medical School	Italy	2000-2002	160
Pettersen, 2017^[19]^	Department of Cardiothoracic Surgery, St. Olavs University Hospital	Norway	2013-2014	100
Samano, 2015^[5]^	Orebro University	Sweden	1993-1997	104
Santos, 2002^[20]^	University of São Paulo	Brazil	1998-1999	60
Song, 2012^[21]^	Yonsei University College of Medicine	Korea	2008-2009	60
Tian, 2021^[3]^	Multicenter	China	2017-2019	2655
Toure, 2021^[10]^	Kasr el Ainy and Faculty of Medicine Cairo University	Egypt	NR	50

NR=not reported; RAPCO=Radial Artery Patency and Clinical Outcomes; RAPS=Radial Artery Patency Study; RSVP=Radial Artery *versus* Saphenous Vein Patency; SAVE-RITA=Saphenous Vein *versus* Right Internal Thoracic Artery

**Table S2:** Inclusion and exclusion criteria of the included trials.

Author, year	Key Inclusion/exclusion criteria	Cohort description
Angelini, 2021^[8]^	Inclusion: adults aged 18 years and over undergoing first time CABG (either on- or off-pump) with at least one saphenous vein graft. Exclusion: valve replacement/repair or an aortic procedure, congestive heart failure, ejection fraction < 30%, preoperative serum creatinine > 104 µmol/L, peripheral vascular disease, allergy to iodinated contrast media, participating in another interventional study, or unwilling to participate in follow-up.	CON-SV *vs.* NT-SV
Buxton, 2020 (RAPCO)^[2]^	Inclusion: elective isolated CABG patients requiring more than 1 bypass conduit were eligible for the trial. An ejection fraction > 35% and at least 1 non-LAD vessel with a proximal stenosis of at least 70% and diameter of at least 1.5 mm. The RITA group included patients aged < 70 years (or < 60 years and diabetic) with multivessel CAD requiring at least two grafts. The SVG group included patients aged > 70 (or > 60 years and diabetic) with multivessel CAD requiring at least two grafts. Exclusion: at the surgeons’ discretion, if they had an unusable conduit, experienced an acute myocardial infarction in < 7 days, were undergoing off-pump surgery, had an unsuitable coronary target, LVEF < 35%, language barrier, resided overseas, body mass index > 35 kg/m2, renal impairment with serum creatinine level > 300 µmol/L, lung disease with a FEV1 < 1 L, and major illnesses (*e.g.*, malignancy) with expected survival < 10 years.	Group 1: RA *vs.* RITA Group 2: RA *vs.* CON-SV
Collins, 2008 (RSVP)^[11]^	Inclusion: ages 40-70 years, undergoing primary isolated CABG. Exclusion: LVEF < 25%, positive Allen’s test, history of Raynauds syndrome or vasculitis, bilateral varicose veins, or any condition that may have affected the safety of follow-up angiography.	RA *vs.* CON-SV
Deb, 2012 (RAPS)^[12]^	Inclusion: patients with a dominant circumflex coronary artery were eligible if they had sequential high-grade lesions in the circumflex and graftable obtuse marginal and posterior descending arteries. Exclusion: patients with a history of vasculitis, Raynaud’s syndrome, bilateral varicose vein stripping, or varicose veins were excluded from the study. (a) renal insufficiency (creatinine > 180 umol/L); (b) severe peripheral vascular disease precluding femoral access; (c) coagulopathy or obligatory uninterrupted use of anticoagulants; (d) known allergy to radiographic contrast media; (e) women of childbearing potential; (f) comorbid illness which precludes the use of follow-up angiography; and (g) geographically inaccessible for follow-up angiography. Patients who developed any of the preoperative exclusion criteria following surgery were excluded from late angiography.	RA *vs.* CON-SV
Deb, 2019 (SUPERIOR SVG)^[13]^	Inclusion: > 18 years old, undergoing non-emergent isolated on- or off-pump CABG with an LVEF > 20%, required at least one SV as part of the revascularization strategy, and had a creatinine clearance at least 20 mL/min or higher. Exclusion: patients were excluded if the SV was unusable due to previous vein stripping or poor quality on preoperative duplex or vein mapping, if the patient had a contraindication to CTA, was pregnant or a female of child-bearing age, allergy to fish oil/fish production and nonmedicinal ingredients of the study product, already taking fish oil supplements regularly, had a congenital or acquired coagulation disorder, or considered excessive risk of wound infection according to the clinical judgement of the site surgical investigators.	CON-SV *vs.* NT-SV
Dreifaldt, 2019^[14]^	Inclusion: Patients with three-vessel CAD. Exclusion: age > 65 years, LVEF 120 µmol/L, use of anticoagulants, coagulopathy, allergy to contrast medium, positive Allen’s test result or an abnormal result of a Doppler study of the arms, a history of vasculitis or Raynaud’s syndrome, bilateral varicose veins, or previous vein stripping.	RA *vs.* NT-SV
Gaudino, 2005^[15]^	Inclusion: patients undergoing primary elective CABG, had undergone previous percutaneous coronary angioplasty with successful stent implantation in any coronary vessel > 1.2 mm in diameter at least 1 month before surgery with preoperative angiographic demonstration of failed or patent intracoronary stent, and angiographic evidence of triple vessel coronary disease with a diseased (proximal stenosis ≥ 70%) graftable (≥ 1 mm in diameter) obtuse marginal artery, LVEF > 50%, and no preoperative evidence or history of lateral or posterolateral myocardial infarction. Exclusion: patients who underwent stent implantation < 1 month before surgery were excluded, in the presumption that stent failure in such limited time frame could be technically related.	RITA *vs.* RA *vs.* CON-SV
Glineur, 2011^[16]^	Inclusion: patients with life expectancy of > 5 years, undergoing elective isolated CABG with angiographic evidence of severe (> 70% by visual estimate) coronary obstruction on the RCA territory with a perioperative lumen diameter of the right GEA > 1.5 mm. Exclusion: a history of upper abdominal surgery, history of upper gastrointestinal bleeding or active gastric/duodenal ulcer, BMI > 35, diabetes with hemoglobin A1c > 7.5, FEV1 < 60% predicted, redo surgery, cirrhosis, or other configuration than graft to posterior descending artery or posterior lateral artery.	RA *vs.* right GEA
Goldman, 2011^[17]^	Inclusion: patients undergoing elective first-time CABG without concomitant valve procedure. Exclusion: requirement for only a single vessel bypass where the left internal mammary artery would be used for that graft; previous vein stripping and ligation of saphenous veins with no venous conduit available for bypass; Raynaud’s symptoms; creatinine > 2.0 mg/dL or requiring hemodialysis; positive Allen’s test; cardiogenic shock, or unable to give consent; allergic to contrast material; undergoing repeat CABG; less than full use of both arms; currently pregnant; neurologic or musculoskeletal disease affecting the arm; refusal to participate; requirement for any concomitant valve operation in the mitral, aortic, or pulmonary position; isolated tricuspid annuloplasty was acceptable but tricuspid valve replacement excluded the patient from consideration; concomitant Dor or Maze procedure; in another research study; or no suitable radial target (there is no non-LAD vessel with a > 70% stenosis).	RA *vs.* CON-SV
Hou, 2021^[9]^	Inclusion: aged 18-80 years, at least three-vessel CAD, and voluntarily joined the study and signed the informed consent form. Exclusion: simultaneous operations (such as heart valve or lung or abdominal surgery), emergency surgery, ejection fraction ≤ 35%, complicated with interventricular septal perforation and ventricular aneurysm, redo CABG, internal diameter of great saphenous vein ≤ 0.20 cm, varicose great saphenous vein, or venous tortuosity, complicated with severe malignant tumor or other serious systemic diseases, severe renal insufficiency (creatinine > 200 µmol/L), dual antiplatelet taboo, severe peripheral vascular disease, allergy to the radio-contrast agent, participation in other clinical trials at the same time.	CON-SV *vs.* NT-SV
Kim, 2021 (SAVE RITA)^[6]^	Inclusion: patients aged 40-70 years undergoing off-pump CABG for multivessel CABG using a Y-composite graft based on the *in situ* left internal thoracic artery. Exclusion: ineligible Y-composite graft revascularization, an unavailable RITA or SV, LVEF ≤ 25%, chronic renal failure requiring renal replacement therapy, previous cardiac surgery, emergency operation, or a medical history such as malignant disease that might limit the possibility of midterm follow-up.	CON-SV *vs.* RITA
Muneretto, 2004^[18]^	Inclusion: patients aged > 70 years and scheduled for on-pump isolated myocardial revascularization. Exclusion: age < 70 years, single-vessel disease, emergency operations, concomitant procedures other than coronary surgery, LVEF < 20%, EuroSCORE > 10, and the presence of a positive Allen’s test.	RA *vs.* CON-SV
Pettersen, 2017^[19]^	Inclusion: patients undergoing isolated first-time non-emergent CABG requiring cardiopulmonary bypass with an LVEF > 35% with at least one saphenous vein graft required as part of the revascularization strategy. Exclusion: any acute or chronic inflammatory diseases, patient with a history of malignancy, pregnancy, or previous cardiac surgery, serum creatinine > 120 umol/L, coagulopathy, insulin-dependent diabetes, smoking during last 6 months, leg not suitable for no-touch vein harvesting as judged by the operator, need for nitrates on operation day, and patients not on statins.	CON-SV *vs.* NT-SV
Samano, 2015^[5]^	Exclusion: unstable angina, insulin-dependent diabetes mellitus, serum creatinine > 120 umol/L, preventive use of anticoagulants, coagulopathy, combined procedure, redo CABG, and severe peripheral vascular disease.	CON-SV *vs.* NT-SV
Santos, 2002^[20]^	Exclusion: (a) age over 70 years; (b) severe obesity; (c) previous abdominal operation; (d) positive Allen’s test; (e) redo operation; (f) additional procedure; (g) severely depressed left ventricular function; (h) contraindications for use of calcium-channel blockers; and (i) contraindication for postoperative angiography.	RA *vs.* right GEA
Song, 2012^[21]^	Inclusion: age ≥ 70 years and primary isolated off-pump CABG. Exclusion: single-vessel disease, emergent surgery, a positive Allen’s test, or acute or chronic renal failure.	RA *vs.* NT-SV
Tian, 2021^[3]^	Inclusion: patients aged 18 years or older who was planned to undergo primary isolated open-chest CABG with at least one graft from saphenous vein, with or without cardiopulmonary bypass. Exclusion: concomitant cardiac or vascular surgeries (*i.e.*, valve repair or replacement, Maze surgery), redo CABG, emergency CABG, use of vascular stapler for anastomosis, planned endarterectomy of coronary artery during surgery, left ventricular repair due to ventricular aneurysm, malignant tumor or other severe systemic diseases, severe renal insufficiency (*i.e.*, serum creatinine > 200 µmol/L), contraindications for dual antiplatelet therapy, such as active gastroduodenal ulcer, participant of other ongoing clinical trials.	CON-SV *vs.* NT-SV
Toure, 2021^[10]^	Inclusion: target lesion in oblique marginal is proximal and tight (> 80%), LVEF > 40%.	RA *vs.* CON-SV

BMI=body mass index; CABG=coronary artery bypass grafting; CAD=coronary artery disease; CON-SV=conventionally harvested saphenous vein; CTA=computed tomography angiography; EuroSCORE=European System for Cardiac Operative Risk Evaluation; FEV1=forced expiratory volume in 1 second; GEA=gastroepiploic artery; LAD=left anterior descending; LVEF=left ventricular ejection fraction; NT-SV=no-touch saphenous vein; RA=radial artery; RAPCO=Radial Artery Patency and Clinical Outcomes; RAPS=Radial Artery Patency Study; RCA=right coronary artery; RITA=right internal thoracic artery; RSVP=Radial Artery *versus* Saphenous Vein Patency; SAVE-RITA=Saphenous Vein *versus* Right Internal Thoracic Artery; SV=saphenous vein; SVG=saphenous vein graft

A total of 6,543 randomized patients were included in the final analysis. Demographics of the included patients are presented in Table S3. The number of patients in the trials ranged from 50 to 2,655. The mean age range was 58.0 to 76.9 years in the CON-SV group, 61.0 to 77.6 years in the NT-SV group, 55.7 to 77.3 years in the RA group, 59.5 to 63.5 years in the RITA group, and 56.1 to 61.9 years in the GEA group. Female patients ranged from 1% to 46% in the CON-SV group, 7% to 44% in the NT-SV group, 0% to 51% in the RA group, 5% to 19% in the RITA group, and 12% to 13% in the GEA group. The prevalence of diabetes mellitus ranged from 4% to 84% in the CON-SV group, 2% to 76% in the NT-SV group, 11% to 49% in the RA group, were 11% in the RITA group, and ranged from 20% to 27% in the GEA group. The details of procedure- and angiography-related variables are shown in Tables S4 and S5, respectively.

**Table S3:** Demographics of included patients.

Author, year	Age (Mean ± SD)	Sex (Female), N (%)	Hypertension, N (%)	Diabetes, N (%)	Dyslipidemia, N (%)
Angelini, 2021^[8]^	CON-SV: 65.0 ± 8.6 NT-SV: 67.6 ± 7.3	CON-SV: 4.3 NT-SV: 15.4	CON-SV: 82.6 NT-SV: 73.1	CON-SV: 8.7 NT-SV: 19.2	CON-SV: 100 NT-SV: 88.5
Buxton, 2020 (RAPCO-RITA)^[2]^	RA: 59.2 RITA: 59.5	RA: 12.0 RITA: 9.0	RA: 57.0 RITA: 51.0	RA: 11.0 RITA: 11.0	NR
Buxton, 2020 (RAPCO-SV)^[2]^	RA: 72.6 CON-SV: 73.1	RA: 19.0 CON-SV: 19.0	RA: 60.0 CON-SV: 70.0	RA: 44.0 CON-SV: 46.0	NR
Collins, 2008 (RSVP)^[11]^	RA: 58.0 ± 6.0 CON-SV: 58.0 ± 8.0	RA: 3.0 CON-SV: 5.0	RA: 58.0 CON-SV: 50.0	RA: 19.0 CON-SV: 14.0	RA: 69.0 CON-SV: 84.0
Deb, 2012 (RAPS)^[12]^	RA: 60.4 ± 8.0 CON-SV: 60.4 ± 8.0	RA: 15.2 CON-SV: 15.2	RA: 45.0 CON-SV: 45.0	RA: 30.9 CON-SV: 30.9"	RA/CON-SV: 70.3
Deb, 2019 (SUPERIOR SVG) ^[13]^	CON-SV: 64.0 ± 8.2 NT-SV: 65.5 ± 9.0	CON-SV: 8.1 NT-SV: 16.5	CON-SV: 83.7 NT-SV: 75.6	CON-SV: 83.7 NT-SV: 75.6"	NR
Dreifaldt, 2019^[14]^	Overall: 59.0	Overall: 12.0	Overall: 50.0	Overall: 18.0	Overall: 89.0
Gaudino, 2005 (control)^[15]^	Overall: 63.0 ± 8.0	Overall: 29.0	Overall: 21.0	Overall: 22.0	Overall: 35.0
Gaudino, 2005 (study)^[15]^	Overall: 65.0± 9.0	Overall: 25.0	Overall: 18.0	Overall: 40.0	Overall: 38.0
Glineur, 2011^[16]^	CON-SV: 63.1 ± 7.7 RITA: 62.9 ± 8.3 GEA: 61.9 ± 8.3	CON-SV: 6.0 RITA: 5.0 GEA: 12.0	CON-SV: 76.0 RITA: 28.0 GEA: 82.0	CON-SV: 24.0 RITA: 11.0 GEA: 27.0	CON-SV: 71.0 RITA: 27.0 GEA: 82.0
Goldman, 2011^[17]^	RA: 61.0 ± 8.0 CON-SV: 62.0± 8.0	RA: 0.0 CON-SV: 1.0	RA: 79.0 CON-SV: 79.0	RA: 42.0 CON-SV: 42.0	NR
Hou, 2021^[9]^	CON-SV: 59.8 ± 7.8 NT-SV: 61.0 ± 8.7	CON-SV: 6.0 NT-SV: 8.0	CON-SV: 60.0 NT-SV: 58.0	CON-SV: 40.0 NT-SV: 36.0	CON-SV: 22.0 NT-SV: 24.0
Kim, 2021 (SAVE-RITA)^[6]^	CON-SV: 64 RITA: 63.5	CON-SV: 24.8 RITA: 19.1	NR	NR	NR
Muneretto, 2004^[18]^	RA: 77.3 ± 3.0 CON-SV: 76.9 ± 2.0	RA: 43.7 CON-SV: 46.2	NR	RA: 48.7 CON-SV: 45.0	NR
Pettersen, 2017^[19]^	CON-SV: 65.0 ± 6.9 NT-SV: 63.4 ± 7.1	CON-SV: 18.0 NT-SV: 7.0	NR	CON-SV: 4.0 NT-SV: 2.0	NR
Samano, 2015^[5]^	CON-SV: 71.4 NT-SV: 77.6	CON-SV: 14.8 NT-SV: 7.4	CON-SV: 67.0 NT-SV: 56.0	CON-SV: 30.0 NT-SV: 37.0"	CON-SV: 93.0 NT-SV: 96.0
Santos, 2002^[20]^	RA: 55.7 ± 7.9 GEA: 56.1 ± 7.7	RA: 16.7 GEA: 13.3	RA: 70.0 GEA: 80.0	RA: 26.7 GEA: 20.0	NR
Song, 2012^[21]^	RA: 72.7 ± 3.5 NT-SV: 74.6 ± 3.8	RA: 51.4 NT-SV: 44	RA: 65.7 NT-SV: 84.0	RA: 42.9 NT-SV: 52.0	RA: 48.6 NT-SV: 44.0
Tian, 2021^[3]^	CON-SV: 60.8 ± 8.0 NT-SV: 60.9 ± 8.4	CON-SV: 21.8 NT-SV: 21.4	CON-SV: 61.8 NT-SV: 64.5	CON-SV: 35.1 NT-SV: 36.2	CON-SV: 69.2 NT-SV: 68.0
Toure, 2021^[10]^	NR	NR	NR	NR	NR

CON-SV=conventionally harvested saphenous vein; GEA=gastroepiploic artery; NR=not reported; NT-SV=no-touch saphenous vein; RA=radial artery; RAPCO=Radial Artery Patency and Clinical Outcomes; RAPS=Radial Artery Patency Study; RITA=right internal thoracic artery; RSVP=Radial Artery *versus* Saphenous Vein Patency; SAVE-RITA=Saphenous Vein *versus* Right Internal Thoracic Artery; SD=standard deviation; SV=saphenous vein

**Table S4:** Procedure-related variables by trial.

Author, year	Graft to circumflex coronary system (%)	Proximal anastomosis to ascending aorta (%)	Off-pump CABG (%)
Angelini, 2021^[8]^	CON-SV: 40.5 NT-SV: 45.7	NR	CON-SV: 69.6 NT-SV: 57.7
Buxton, 2020 (RAPCO-RITA)^[2]^	RA: 62 RITA: 67	RA: 100 RITA: 100	RA: 0 RITA: 0
Buxton, 2020 (RAPCO-SV)^[2]^	RA: 68 CON-SV: 60	RA: 100 CON-SV: 100	RA: 0 CON-SV: 0
Collins, 2008 (RSVP)^[11]^	NR	"RA: 100 CON-SV: 100"	"RA: 0 CON-SV: 0"
Deb, 2012 (RAPS)^[12]^	RA: 50 CON-SV: 50	RA: 98.4 CON-SV: 99.6	NR
Deb, 2019 (SUPERIOR SVG)^[13]^	NR	NR	NR
Dreifaldt, 2019^[14]^	RA: 63 NT-SV:62	NR	RA: 0 NT-SV: 0
Gaudino, 2005 (control)^[15]^	RA: 100 CON-SV: 100 RITA: 100	RA: 100 CON-SV: 100 RITA: 100	RA: 0 CON-SV: 0 RITA: 0
Gaudino, 2005 (study)^[15]^	RA: 100 CON-SV: 100 RITA: 100	RA: 100 CON-SV: 100 RITA: 100	RA: 0 CON-SV: 0 RITA: 0
Glineur, 2011^[16]^	CON-SV: 0 RITA: 0 GEA: 0	CON-SV: 100 RITA: 0 GEA: 100	NR
Goldman, 2011^[17]^	RA: 55 CON-SV: 59	RA: 100 CON-SV: 100	RA: 11 CON-SV: 13
Hou, 2021^[9]^	NR	NR	CON-SV: 100 NT-SV: 100
Kim, 2021 (SAVE RITA)^[6]^	CON-SV: 99.2 RITA: 96.6	CON-SV: 0 RITA: 0	CON-SV: 100 RITA: 100
Muneretto, 2004^[18]^	RA: 50 CON-SV: 52	RA: 0 CON-SV: 0	RA: 0 CON-SV: 0
Pettersen, 2017^[19]^	NR	CON-SV: 100 NT-SV: 100	CON-SV: 0 NT-SV: 0
Samano, 2015^[5]^	CON-SV: 62 NT-SV: 78	CON-SV: 100 NT-SV: 100	NR
Santos, 2002^[20]^	RA: 55 GEA: 55	RA: 0 GEA: 0	RA: 0 GEA: 0
Song, 2012^[21]^	NR	RA: 0 NT-SV: 0	RA: 100 NT-SV: 100
Tian, 2021^[3]^	CON-SV: 27.1 NT-SV: 27.0	NR	CON-SV: 56.4 NT-SV: 58
Toure, 2021^[10]^	RA: 100 CON-SV: 100	RA: 0 CON-SV: 100	NR

CABG=coronary artery bypass grafting; CON-SV=conventionally harvested saphenous vein; GEA=gastroepiploic artery; NR=not reported; NT-SV=no-touch saphenous vein; RA=radial artery; RAPCO=Radial Artery Patency and Clinical Outcomes; RAPS=Radial Artery Patency Study; RITA=right internal thoracic artery; RSVP=Radial Artery *versus* Saphenous Vein Patency; SAVE-RITA=Saphenous Vein *versus* Right Internal Thoracic Artery; SV=saphenous vein

**Table S5:** Angiography-related variables by trial.

Author, year	Definition of graft occlusion	Number of patients who underwent angiography	Method of angiography	Severity of coronary blockage
Angelini, 2021^[8]^	NR	36	IVUS or catheter-based angiogram	NR
Buxton, 2020 (RAPCO-RITA)^[2]^	1. Total occlusion 2. Stenosis > 80% 3. “String sign” (indicating the absence of functional flow in an arterial graft despite anatomic patency)	326	Catheter-based angiography in 80% of grafts CTA in 20% of grafts	> 70%
Buxton, 2020 (RAPCO-SVG)^[2]^	1. Total occlusion 2. Stenosis > 80%	156	Catheter-based angiography in 82% of grafts CTA in 18% of grafts	> 70%
Collins, 2008 (RSVP)^[11]^	Absence of visible opacification of the study graft despite aortogram. Additional secondary angiographic visual grading of the grafts was defined as P1 = perfect patency; P2 = compromised flow states (stenosis at the anastomoses or in the body of the graft) 50%; P3 = compromised flow states > 50%; P4 = severe diffuse graft narrowing (string sign); and P5 = total occlusion	103	Catheter-based angiography	> 70%
Deb, 2012 (RAPS)^[12]^	Lack of TIMI flow 3	269	Catheter-based angiography in 87% of patients CTA in 13% of patients	> 70%
Deb, 2019 (SUPERIOR SVG)^[13]^	1. Primary outcome: complete occlusion at 1 year 2. Secondary outcomes: significant (50-99%) stenosis, and a composite of significant stenosis or complete occlusion	212	CTA	> 50%
Dreifaldt, 2019^[14]^	No opacification of graft on CTA	99	CTA	> 50%
Gaudino, 2005^[15]^	Four subgroups of patency: 1. Perfectly patent 2. Patent with irregularity 3. Stringed 4. Occluded	120	Catheter-based angiography	> 50% for ISR and > 70% for proximal native stenosis
Glineur, 2011^[16]^	Graft functionality was scored as 0, for an occluded graft; 1, when the flow from the native coronary artery was dominant; 2, when flow supply from the native coronary and the graft was balanced; 3, when the native coronary was fully opacified by the graft; and 4, when the native coronary was fully opacified by the graft only (occluded or sub-occluded coronary native vessel). A graft was considered “not functional” with patency scores of 0 to 2 and “functional” with patency scores of 3 or 4	210	Catheter-based angiography	< 48%, 48-64%, 65-99%, 100%
Goldman, 2011^[17]^	Opacification of distal target by injection of the graft	535	Catheter-based angiography	> 70%
Hou, 2021^[9]^	FitzGibbon-A/B was used for patency, and FitzGibbon-O was used for graft failure	97	CTA	NR
Kim, 2021 (SAVE RITA)^[6]^	FitzGibbon classification: grades A (excellent graft) and B (fair) were considered patent. Grade O (anastomosis), which included stenosis of 75% or more of the grafted coronary artery or a totally occluded graft, was considered occluded	155	"Catheter-based angiography in 60.6% of patients CTA in 39.4% of patients"	> 75%
Muneretto, 2004^[18]^	FitzGibbon classification, that is, grade A (unimpaired graft run-off), grade B (reduced graft caliber < 50% of the grafted coronary artery), and grade C (occluded graft)	136	CTA	> 70% for RA grafts > 60% for ITA grafts
Pettersen, 2017^[19]^	NR	44	Catheter-based angiography	NR
Samano, 2015^[5]^	A graft was judged as occluded when the graft was not opacified by contrast media. A graft stenosis was judged insignificant when the narrowing of the lumen diameter was > 50% relative to the adjacent parts of the vessel	54	CTA	NR
Santos, 2002^[20]^	1. Functioning: good flow, good diameter, filling of the target coronary artery 2. Non-functioning: severe and diffuse spasm and narrowed graft (string sign) or occluded without filling of the target coronary artery	58	Catheter-based angiography	> 75%
Song, 2012^[21]^	NR	190	CTA	NR
Tian, 2021^[3]^	Graft occlusion was considered when a conduit did not fill with contrast at all or string sign was found in any segment. For sequential anastomosis, 1 occlusion of any of the distal anastomoses was judged as occlusion of the whole graft vessel	2434	CTA	< 70%, 70-8%, ≥ 90%
Toure, 2021^[10]^	NR	50	CTA	> 80%

CTA=computed tomography angiography; ISR=in-stent restenosis; ITA=internal thoracic artery; IVUS=intravascular ultrasound; NR=not reported; RA=radial artery; RAPCO=Radial Artery Patency and Clinical Outcomes; RAPS=Radial Artery Patency Study; RITA=right internal thoracic artery; RSVP=Radial Artery *versus* Saphenous Vein Patency; SAVE-RITA=Saphenous Vein *versus* Right Internal Thoracic Artery; SVG=saphenous vein graft; TIMI=thrombolysis in myocardial infarction

A total of 8,272 grafts were analyzed across the 18 included trials: 3,732 CON-SV grafts, 2,647 NT-SV grafts, 1,223 RA grafts, 549 RITA grafts, and 121 GEA grafts. The weighted mean angiographic follow-up time was 3.5 years (95% CI 1.5-5.4). The crude patency rates of the analyzed conduits were as follows: RA 94.1% (95% CI 90.0-97.6); NT-SV 91.4% (95% CI 87.3-94.3); RITA 89.2% (95% CI 71.2-96.5); CON-SV 86.3% (95% CI 81.2-90.2); and GEA 61.2% (95% CI 52.2-69.4). Details of patency rates are given in Table 2.

**Table 2:** Pooled patency of different grafts.

Graft	Number of studies	Number of grafts	Pooled patency rate (95% CI)	Pooled angiographic follow-up (years)
RA	11	1223	94.1 (90.0 - 97.6)	5.46
NT-SV	8	2647	91.4 (87.3 - 94.3)	1.85
RITA	5	549	89.2 (71.2 - 96.5)	6.98
CON-SV	15	3732	86.3 (81.2 - 90.2)	2.85
GEA	2	121	61.2 (52.2 - 69.4)	2.89

CI=confidence interval; CON-SV=conventionally harvested saphenous vein; GEA=gastroepiploic artery; NT-SV=no-touch saphenous vein; RA=radial artery; RITA=right internal thoracic artery

**Fig. 1:**
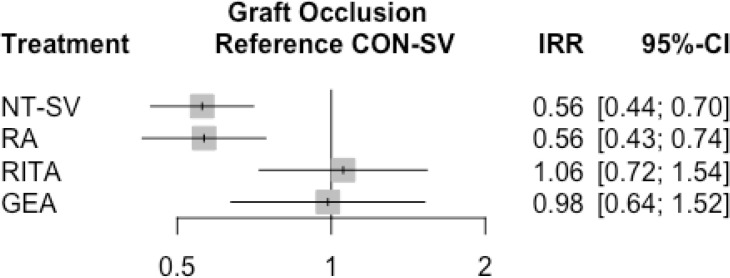
Forest plot for graft occlusion for the different conduits. CI=confidence interval; CON-SV=conventionally harvested saphenous vein; GEA=gastroepiploic artery; IRR=incidence rate ratio; NT-SV=no-touch saphenous vein; RA=radial artery; RITA=right internal thoracic artery.

**Fig. 2:**
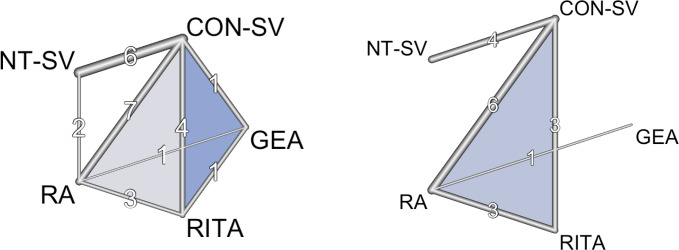
Net graph of the different comparisons for A) the primary outcome of graft occlusion and B) the secondary outcome of late mortality. Width of the lines indicate the number of studies comparing each pair of treatment. In the network plots, colored polygons indicate the presence of multi-arm (3 or more) trials, whereas line shading and thickness are inversely proportional to standard errors of the fixed effect estimate stemming from direct between-arm comparisons. CON-SV=conventionally harvested saphenous vein; GEA=gastroepiploic artery; NT-SV=no-touch saphenous vein; RA=radial artery; RITA=right internal thoracic artery.

**Fig. S2:**
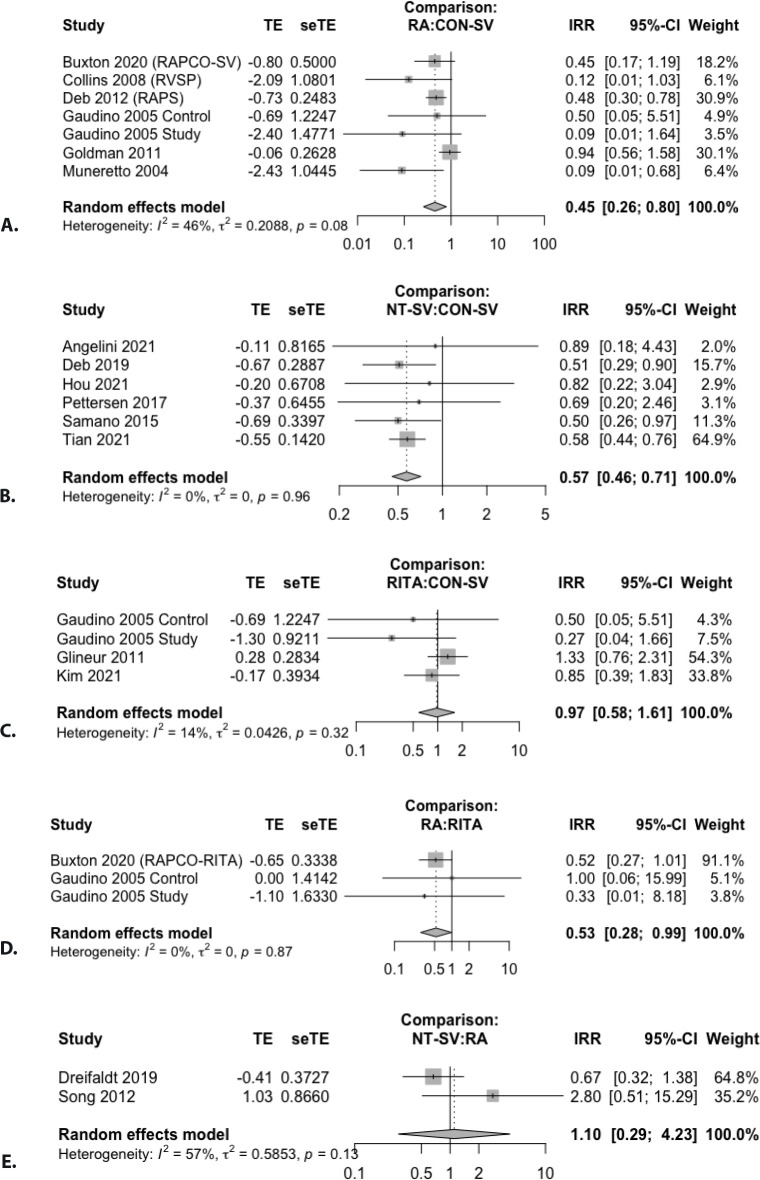
Forest plot for the pairwise comparison of graft occlusion for A) radial artery (RA) vs. conventionally harvested saphenous vein (CON-SV); B) no-touch saphenous vein (NT-SV) vs. CON-SV; C) right internal thoracic artery (RITA) vs. CON-SV; D) RA vs. RITA; E) and NT-SV vs. RA. CI=confidence interval; IRR=incidence rate ratio; RAPCO=Radial Artery Patency and Clinical Outcomes; RAPS=Radial Artery Patency Study; RSVP=Radial Artery Versus Saphenous Vein Patency; SAVE-RITA=Saphenous Vein versus Right Internal Thoracic Artery; seTE=standard error of treatment estimate; SV=saphenous vein; TE=estimate of treatment effect, e.g., log hazard ratio or risk difference.

With CON-SV as reference, only RA (incidence rate ratio [IRR] 0.56; 95% CI 0.43-0.74) and NT-SV (IRR 0.56; 95% CI 0.44-0.70) were associated with significantly lower rate of graft occlusion, whereas RITA (IRR 1.06; 95% CI 0.73-1.54) and GEA (IRR 0.98; 95% CI 0.64-1.52) were not (Table 3, Figure 1, Figure 2A). The width of the CI supports a clinically meaningful benefit of RA and NT-SV in comparison to CON-SV. NT-SV was ranked as the best conduit with a rank score of 0.88 *vs.* 0.87 for RA, 0.29 for GEA, 0.27 for CON-SV, and 0.20 for RITA. These results were confirmed in the individual pairwise meta-analyses (Figure S2 and Table S6A).

**Table 3:** League tables summarizing the results of the network meta-analysis (expressed as incidence rate ratio with 95% confidence interval) for graft occlusion using random effects model.

Graft occlusion				
CON-SV				
**1.79 (1.42** - **2.25)**	NT-SV			
**1.77 (1.34** - **2.34)**	0.99 (0.71 - 1.39)	RA		
0.95 (0.65 - 1.38)	**0.53 (0.34** - **0.82)**	**0.53 (0.36** - **0.80)**	RITA	
1.02 (0.66 - 1.57)	**0.57 (0.35** - **0.93)**	**0.57 (0.35** - **0.93)**	1.07 (0.66 - 1.73)	GEA

CON-SV=conventionally harvested saphenous vein; GEA=gastroepiploic artery; NT-SV=no-touch saphenous vein; RA=radial artery; RITA=right internal thoracic artery

**Fig. S3:**
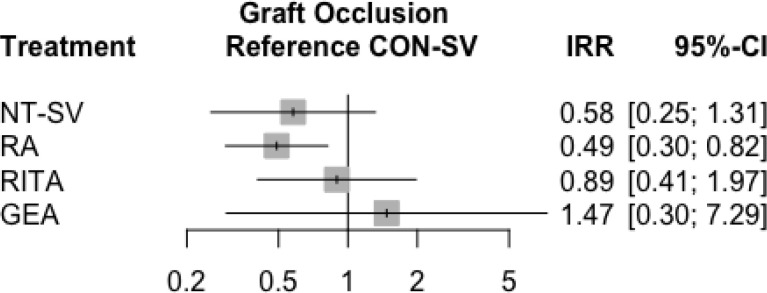
Subgroup analysis for the primary outcome in studies with target vessel stenosis ≥ 70%. CI=confidence interval; CON-SV=conventionally harvested saphenous vein; GEA=gastroepiploic artery; IRR=incidence rate ratio; NT-SV=no-touch saphenous vein; RA=radial artery; RITA=right internal thoracic artery.

**Fig. S4:**
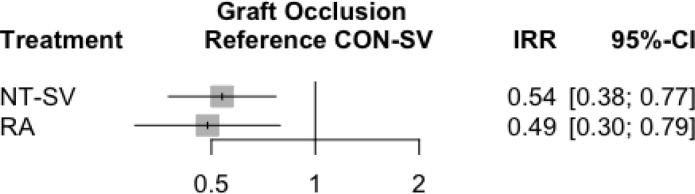
Sensitivity analyses for studies using computed tomography angiography exclusively for postoperative graft assessment. There were not enough studies reporting data for the right internal thoracic artery and the gastroepiploic artery. CI=confidence interval; CON-SV=conventionally harvested saphenous vein; IRR=incidence rate ratio; NT-SV=no-touch saphenous vein; RA=radial artery.

The results of the sensitivity analysis for target vessel stenosis ≥ 70% showed superiority of RA (IRR, 0.49; 95% CI, 0.30-0.82) to CON-SV, but no significant difference between NT-SV (IRR, 0.58; 95% CI, 0.25-1.31) and CON-SV (Figure S3). Studies using CTA for graft assessment were consistent with the primary analysis (Figure S4).

**Fig. 3:**
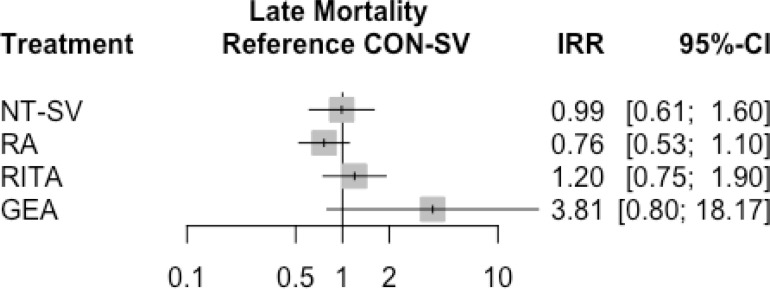
Forest plot for late mortality for the different conduits. CI=confidence interval; CON-SV=conventionally harvested saphenous vein; GEA=gastroepiploic artery; IRR=incidence rate ratio; NT-SV=no-touch saphenous vein; RA=radial artery; RITA=right internal thoracic artery.

Late mortality was comparable between conduits at a weighted mean follow-up time of 3.5 years (Figures 2B and 3, Tables 4 and S6B). The network RA *vs.* GEA comparison appeared to favor RA, with limited data - although only one study directly compared the two conduits^[^20^]^.

**Table 4:** League tables summarizing the results of the network meta-analysis (expressed as incidence rate ratio with 95% confidence interval) for late mortality using random effects model.

Late mortality				
CON-SV				
1.01 (0.63 - 1.63)	NT-SV			
1.31 (0.91 - 1.90)	1.30 (0.71 - 2.38)	RA		
0.84 (0.53 - 1.33)	0.83 (0.42 - 1.61)	0.64 (0.41 - 1.00)	RITA	
0.26 (0.06 - 1.25)	0.26 (0.05 - 1.33)	**0.20 (0.04** - **0.91)**	0.31 (0.06 - 1.53)	GEA

CON-SV=conventionally harvested saphenous vein; GEA=gastroepiploic artery; NT-SV=no-touch saphenous vein; RA=radial artery; and RITA=right internal thoracic artery

**Table S6:** Summary of different pairwise comparisons using random effects modeling for A) graft occlusion and B) late mortality. For each pairwise comparison, the second group is the reference arm.

A.					
**Outcomes**	**Studies**	**IRR (95% CI)**	**I2**	**Heterogeneity *P*-value**	**Overall effect *P*-value**
Graft occlusion					
RA *vs.* CON-SV	7	0.45 (0.26 - 0.80)	0.46	0.08	0.01
NT-SV *vs.* CON-SV	6	0.57 (0.46 - 0.72)	0.0	0.96	< 0.0001
RITA *vs.* CON-SV	4	0.97 (0.58 - 1.60)	0.14	0.32	0.91
RA *vs.* RITA	3	0.53 (0.28 - 0.99)	0.0	0.87	47
NT-SV *vs.* RA	2	0.83 (0.43 - 1.63)	0.57	0.13	0.88
**B.**					
**Outcomes**	**Studies**	**IRR (95% CI)**	**I2**	**Heterogeneity *P*-value**	**Overall effect *P*-value**
Late mortality					
RA *vs.* CON-SV	6	0.81 (0.54 - 1.22)	0.00	1.00	0.32
NT-SV *vs.* CON-SV	4	0.99 (0.61 - 1.60)	0.00	0.46	0.96
RITA *vs.* CON-SV	3	1.04 (0.56 - 1.92)	0.00	1.00	0.90
RA *vs.* RITA	3	0.57 (0.32 - 1.00)	0.00	0.92	0.05

CI=confidence interval; CON-SV=conventionally harvested saphenous vein; IRR=incidence rate ratio; NT-SV=no-touch saphenous vein; RA=radial artery; RITA=right internal thoracic artery

**Fig. S5:**
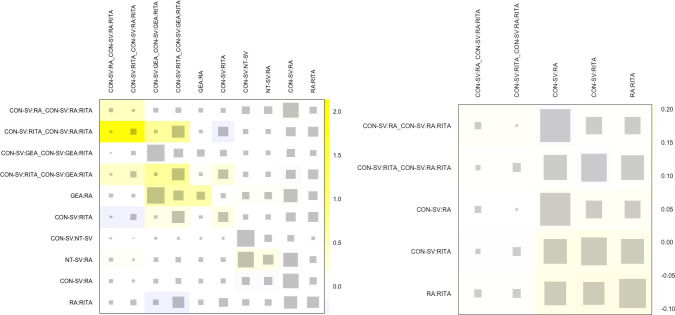
Net heat plot evaluating for inconsistency (i.e., disagreement between direct and indirect evidence) in the network model for A) graft patency and B) late mortality. The areas of gray squares represent the relative contributions of designs listed in the columns to the network estimate of designs listed in the rows. The colors are associated with changes in inconsistency between direct and indirect evidence in designs listed in the rows after detaching the effect of designs listed in the columns. Yellow colors indicate a decrease (the stronger the intensity of the color, the stronger the change). CON-SV=conventionally harvested saphenous vein; GEA=gastroepiploic artery; NT-SV=no-touch saphenous vein; RA=radial artery; RITA=right internal thoracic artery.

Heterogeneity/inconsistency estimates and net split are shown in Tables S7 and S8, and in the net heat plot shown in Figure S5. Overall heterogeneity was low (I^^2^^ < 5%) for graft patency and late mortality (Table S8). Risk of bias was low for most of the trials (Table 5).

**Table S7:** Assessment of inconsistency based on separate indirect from direct evidence (or SIDE) using back-calculation method and random effects model.

Graft occlusion	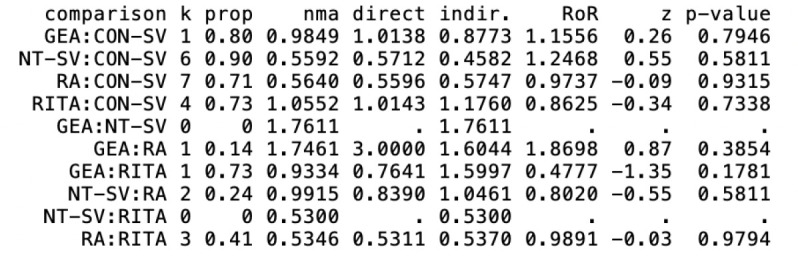
Late mortality	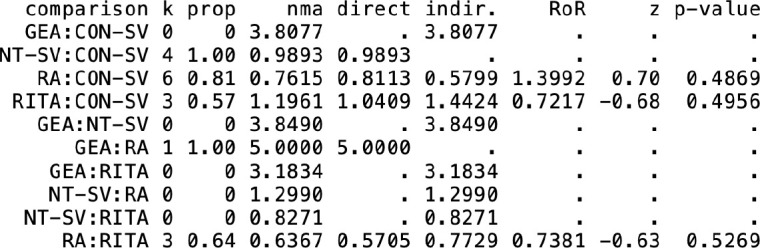

All *P*-values were insignificant reflecting no significant disagreement (no inconsistency) between the direct and indirect estimate in our included outcomes.

CON-SV=conventionally harvested saphenous vein; GEA=gastroepiploic artery; NT-SV=no-touch saphenous vein; RA=radial artery; RITA=right internal thoracic artery

In this table: comparison=treatment comparison; k=number of studies providing direct evidence; prop=direct evidence proportion; nma=estimated treatment effect (incidence rate ratio [IRR]) in network meta-analysis; direct=estimated treatment effect (IRR) derived from direct evidence; indir.=estimated treatment effect (IRR) derived from indirect evidence; RoR=ratio of ratios (direct *vs.* indirect); z=*z*-value of test for disagreement (direct *vs.* indirect); p-value=*P*-value of test for disagreement (direct *vs.* indirect)

**Table S8:** Quantifying heterogeneity.

Outcome	Quantifying heterogeneity/inconsistency	Tests of heterogeneity (within designs) and inconsistency (between designs)
Graft occlusion	Tau^^2^^ = 0.0052, I^^2^^ = 2.9%	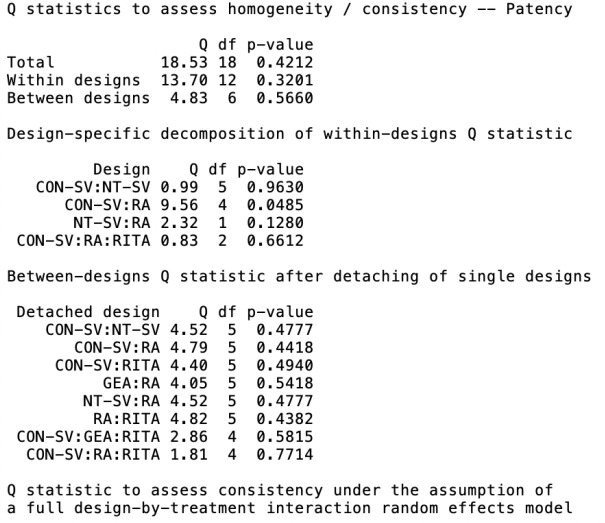
Late mortality	Tau^^2^^ = 0, I^^2^^ = 0%	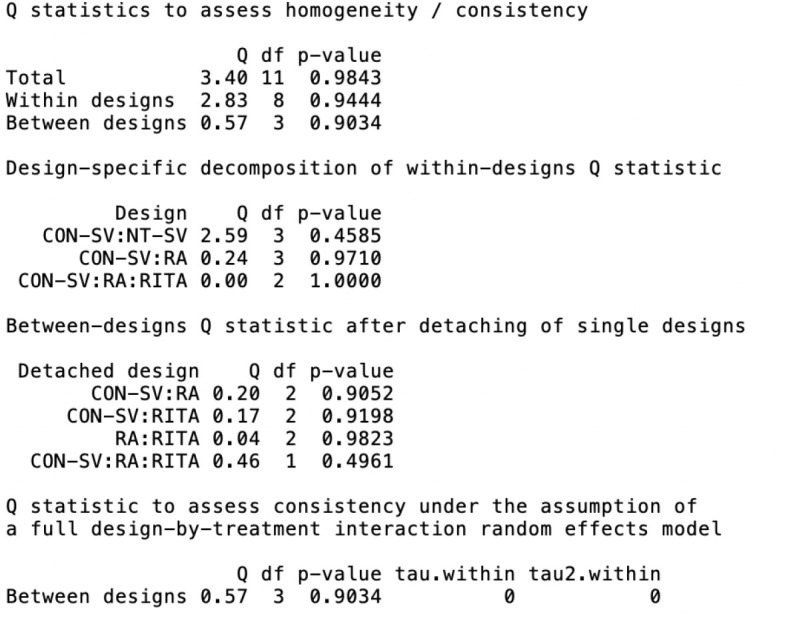

Tests of heterogeneity (within designs) and inconsistency (between designs) and design-specific decomposition of within-designs Q statistic. CON-SV=conventionally harvested saphenous vein; GEA=gastroepiploic artery; NT-SV=no-touch saphenous vein; RA=radial artery; RITA=right internal thoracic artery

**Table 5:** Assessment of risk of bias using the Cochrane Collaboration’s tool.

Author, year	Random Sequence Generation	Allocation Concealment	Blinding of Participants	Blinding of Outcome Assessment	Incomplete Outcome Data	Selective reporting
Angelini, 2021^[8]^	+	+	+	+	+	+
Buxton, 2020 (RAPCO)^[2]^	+	+	-	+	+	+
Collins, 2008 (RSVP)^[11]^	+	+	+	+	+	-
Deb, 2012 (RAPS)^[12]*^	+	-	-	+	+	-
Deb, 2019 (SUPERIOR SVG)^[13]^	+	+	+	+	+	+
Dreifaldt, 2019^[14]*^	+	-	-	+	+	+
Gaudino, 2005^[15]^	+	?	-	+	+	+
Glineur, 2011^[16]^	+	+	-	+	?	+
Goldman, 2011^[17]^	+	+	?	?	+	+
Hou, 2021^[9]^	+	+	+	?	+	+
Kim, 2021 (SAVE-RITA)^[6]^	+	-	+	+	+	+
Muneretto, 2004^[18]^	+	-	?	+	+	+
Pettersen, 2017^[19]^	+	?	?	+	?	?
Samano, 2015^[5]^	+	-	+	+	+	+
Santos, 2002^[20]^	+	-	-	+	+	+
Song, 2012^[21]^	+	+	?	+	+	+
Tian, 2021^[3]^	+	+	+	+	+	+
Toure, 2021^[10]^	?	?	?	?	+	?

RAPCO=Radial Artery Patency and Clinical Outcomes; RAPS=Radial Artery Patency Study; RSVP=Radial Artery *versus* Saphenous Vein Patency; SAVE-RITA=Saphenous Vein versus Right Internal Thoracic Artery

^*^For Deb, 2012 and Dreifaldt, 2019, every patient received both study grafts. However, the endpoint assessors were blinded

Green=low risk; yellow=uncertain risk; red=high risk

**Fig. S6:**
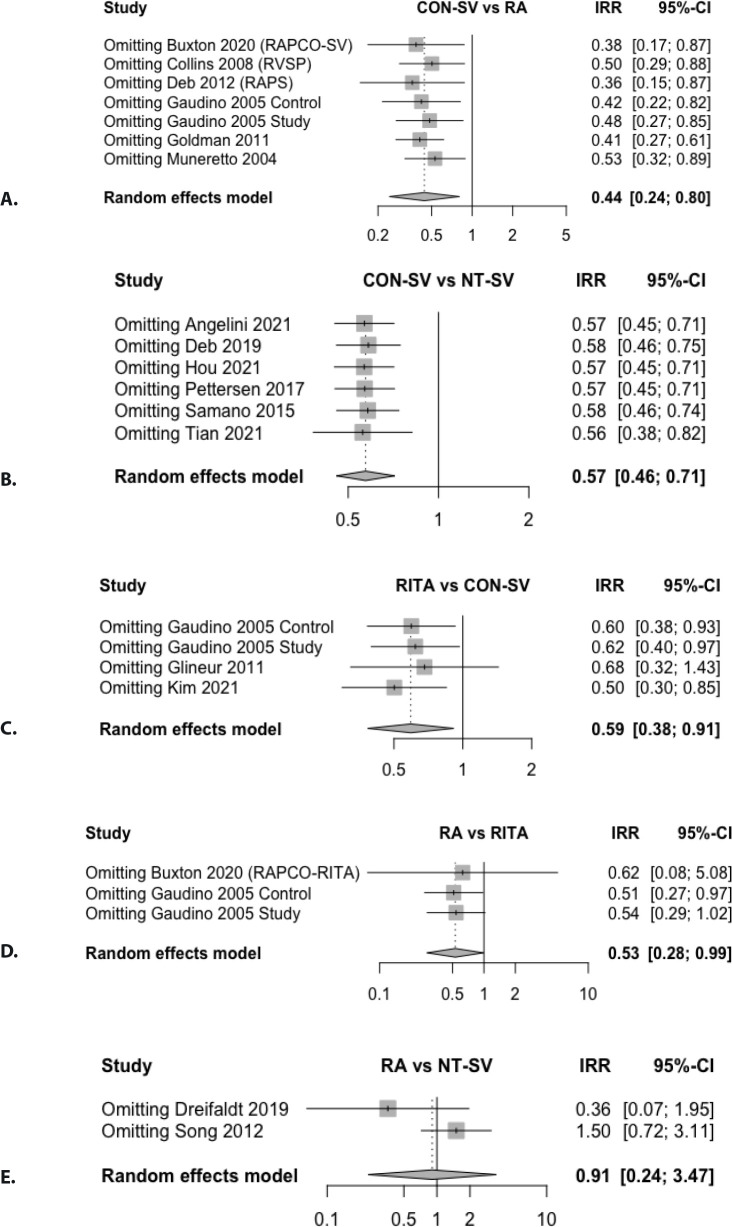
Leave-one-out analysis for graft occlusion in A) radial artery (RA) vs. conventionally harvested saphenous vein (CON-SV); B) no-touch saphenous vein (NT-SV) vs. CON-SV; C) right internal thoracic artery (RITA) vs. CON-SV; D) RA vs RITA; E) RA vs. NT-SV. CI=confidence interval; IRR=incidence rate ratio; RAPCO=Radial Artery Patency and Clinical Outcomes; RAPS=Radial Artery Patency Study; RSVP=Radial Artery Versus Saphenous Vein Patency; SV=saphenous vein.

**Fig. S7:**
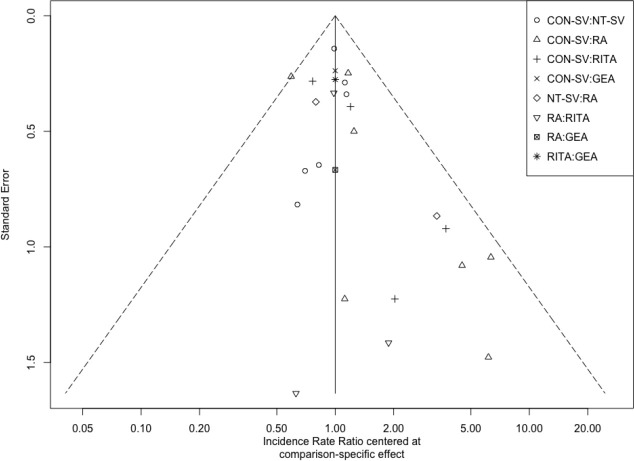
Funnel plot for all studies. CON-SV=conventionally harvested saphenous vein; GEA=gastroepiploic artery; NT-SV=no-touch saphenous vein; RA=radial artery; RITA=right internal thoracic artery.

Leave-one-out analysis and funnel plot did not identify strong evidence of invalidity of the main analysis (Figures S6 and S7).

### Meta-regression

Comparing RA and CON-SV, the percentage of off-pump technique use was directly associated, and the percentage of female patients was inversely associated with the IRR for the primary outcome of graft occlusion. There was no significant association between the variables and other graft comparisons in the meta-regression (Table S9).

**Table S9:** Meta-regression for the primary outcome of graft occlusion.

	RA *vs.* CON-SV (n=7)	RITA *vs.* CON-SV (n=4)	RA *vs.* RITA (n=3)	NT-SV *vs.* CON-SV (n=6)	RA *vs.* NT-SV (n=2)
Age	-0.05 ± 0.05, *P*=0.36	-	-	-0.01 ± 0.03, *P*=0.67	-
Female sex	**-0.05 ± 0.02, *P*=0.01**	-	-	0.01 ± 0.02, *P*=0.57	-
Hypertension	0.02 ± 0.01, *P*=0.08	-	-	-0.005 ± 0.02, *P*=0.77	-
Diabetes mellitus	0.05 ± 0.03, *P*=0.10	-	-	-0.008 ± 0.02, *P*=0.67	-
Dyslipidemia	-	-	-	-0.005 ± 0.01, *P*=0.63	-
Target vessel stenosis	1.7 ± 1.57, *P*=0.29	0.47 ± 0.46, *P*=0.31	0.48 ± 1.67, *P*=0.77	-	-
Duration of follow-up	-0.02 ± 0.11, *P*=0.89	0.07 ± 0.07, *P*=0.31	0.05 ± 0.34, *P*=0.88	-0.01 ± 0.02, *P*=0.66	-
Completeness of angiographic follow-up	-0.02 ± 0.02, *P*=0.41	-0.02 ± 0.02, *P*=0.27	0.006 ± 0.04, *P*=0.87	0.004 ± 0.009, *P*=0.66	-
Proximal anastomosis on the ascending aorta	0.02 ± 0.01, *P*=0.18	-0.01 ± 0.01, *P*=0.25	-	-	-
Graft to circumflex coronary system	0.002 ± 0.02, *P*=0.9	-0.01 ± 0.01, *P*=0.51	0.003 ± 0.02, *P*=0.87	0.009 ± 0.07, *P*=0.90	-
Off-pump coronary artery bypass grafting	**0.10 ± 0.05, *P*=0.04**	0.01 ± 0.01, *P*=0.25	-0.005 ± 0.03, *P*=0.87	-0.0003 ± 0.009, *P*=0.98	-

All values are expressed as beta ± standard deviation, *P*-value. Positive beta reflects higher incidence rate ratio of the outcome with increased variable value, while negative beta reflects lower incidence rate ratio of the outcome with higher variable value.

CON-SV=conventionally harvested saphenous vein; NT-SV=no-touch saphenous vein; RA=radial artery; RITA=right internal thoracic artery

## DISCUSSION

In this NMA of 18 RCTs (8,272 grafts), we found that compared with CON-SV, RA and NT-SV have significantly lower occlusion rate at a mean weighted follow-up time of 3.5 years. NT-SV and RA ranked as the best conduits, whereas there was no strong evidence for greater patency in RITA and right GEA when compared to CON-SV.

Currently, there is still a lack of consensus on the second best conduit after the LITA to LAD bypass for non-LAD targets. Meta-analysis of angiographic RCTs allows a robust understanding of patency rates of various conduits while minimizing confounding and risk of bias. By amalgamating the randomized trials, a meta-analysis is the highest level of evidence available. Additionally, NMA provides the advantage of facilitating indirect comparisons of multiple interventions, thereby increasing the power of the analysis.

The comparison between NT-SV and CON-SV was assessed by the largest RCT included in our NMA, with 2,655 randomized patients^[^3^]^. Tian et al.^[^3^]^ reported a lower rate of graft occlusion at 12 months compared to CON-SV, with an odds ratio (OR) of 0.56 (95% CI, 0.41-0.76; *P*<0.001); however, there was no difference in major adverse cardiac and cerebrovascular events. The caveat of NT-SV is a higher rate of leg wound surgical intervention at three months of follow-up (OR 2.55; 95% CI, 1.85-3.52; *P*<0.001)^[^3^]^. Deb et al.^[^13^]^ also showed an over two fold increase in the rate of leg infections (*P*<0.01) and more severe infection with NT-SV (*P*=0.004) at 30 days, compared to CON-SV. Due to an increased risk of harvest-site complications, guidelines recommend NT-SV harvest technique only in patients with low risk of wound complications^[^22^]^. The NT-SV received a Class IIa recommendation in the 2018 European Revascularization guidelines^[^23^]^ and was a Best Practice in the 2021 American College of Cardiology/American Heart Association revascularization guidelines^[^22^]^.

Several large RCTs support the long-term patency of RA over CON-SV^[^2^,^11^,^12^]^. The Radial Artery Database International Alliance (RADIAL) database also reported lower 10-year composite outcome of death, myocardial infarction, or repeat revascularization for patients who received RA relative to CON-SV^[^24^]^. Conversely, the Arterial Revascularization Trial (ART) did not find a difference in survival and event-free survival at 10 years among patients randomized to receive RITA^[^25^]^. However, the ART trial is criticized for its high crossover between single and bilateral internal thoracic artery (BITA) groups and confounding from RA use, which may have diminished the clinical benefit of RITA. In an as-treated analysis of the ART trial, non-randomized data showed a meaningful difference in mortality in favor of multiple arterial grafts. The merit of multiple *vs.* single arterial grafting in improving cardiovascular events and death in patients after CABG is currently being investigated in the ROMA trial (Randomized Comparison of the Outcome of Single versus Multiple Arterial Grafts. ClinicalTrials.gov registration number: 1703018094)^[^26^]^.

The use of RA received a Class I indication and is preferred to saphenous vein as the second most important conduit for a significantly stenosed, non-LAD vessel in the 2021 American revascularization guidelines^[^22^]^. Although RA is a versatile graft, calcium channel blockers are routine adjuncts to prevent vasospasm. RA should only be used to bypass severely stenotic target vessels due to the risk of string sign in the setting of competitive flow.

These findings challenge the previously accepted belief that RITA is the natural second conduit of choice due to its biophysiological similarity with LITA. The explanation is multifactorial. Firstly, there are less randomized evidence regarding RITA and CON-SV when compared to RA and CON-SV (three trials including a total of 353 patients for RITA, seven trials including a total of 841 patients for RA). Secondly, the RAPCO trial used RITA as a free graft, which may affect graft patency. Thirdly, BITA surgery is more technically challenging than using RA and LITA, with successful application of RITA reliant on surgeon experience. This may partly explain the 14% crossover from BITA to the single internal thoracic artery in the ART trial^[^25^]^. Even though the ART trial recruited surgeons with over 50 BITA cases of experience, there was still a wide variation of intraoperative BITA conversion rates across surgeons, which highlights the technical demand of successful BITA grafting^[^27^]^.

There were no differences in late mortality for any of the second conduits, including RA, compared to the control saphenous vein graft. The association between graft patency and survival is biologically sound and demonstrated by the five-year results of the RADIAL database, where there is a concordant association between improved patency of RA compared to the control saphenous vein and reduction of myocardial infarction and repeat revascularization^[^28^]^. These results are further substantiated in the RADIAL 10-year extension study’s post-hoc analysis for survival^[^24^]^. In the NMA and pairwise comparisons, survival in RA patients was greater than in RITA patients, but it did not cross the threshold for statistical significance (95% CI of 1.00). The data for RA *vs.* GEA comparison was limited.

In the previous NMA, RA was ranked as the best conduit^[^4^]^. In this updated NMA, the introduction of five additional trials has led NT-SV to achieve a higher patency ranking than RA, albeit by a very small margin. Of the five RCTs, three investigated NT-SV and CON-SV (n=2,805)^[^3^,^8^,^9^]^, one compared RA and CON-SV (n=50)^[^10^]^, and one assessed RITA and RA (n=224)^[^6^]^. The increased sample size in NT-SV and CON-SV enhanced the power of analysis in favor of NT-SV. Many of the newly added trials reported early-term results, which likely inflated pooled saphenous vein patency and decreased the weighted mean follow-up time of the NMA from 5.1 to 3.5 years. In keeping with the 2021 NMA findings^[^4^]^, no conduit provided a statistically significant mortality benefit over CON-SV. Meta-regression for IRR of graft occlusion continued to suggest a positive association with off-pump CABG use (*i.e.*, increased graft occlusion) and inverse association with increased proportion of female patients (*i.e.*, decreased graft occlusion)^[^4^]^.

### Limitations

Limitations of this meta-analysis included a small sample size causing certain pairwise analyses to be underpowered, varying quality of the RCTs included, and no data collected on renal disease, secondary prevention, and antispasmodic therapy, which are additional factors that influence graft patency. It is worthwhile to note that the included studies involving NT-SV grafts used pedicle harvest technique with^[^8^,^19^,^21^]^ or without manual dilatation with a syringe^[^3^,^5^,^9^,^13^,^14^]^. The factorial trial by Angelini et al. involving CON-SV *vs.* NT-SV and low- *vs.* high-pressure graft dilation reported that low-pressure distention of CON-SV can achieve wall thickening comparable to NT-SV^[^8^]^.

## CONCLUSION

In this NMA of 18 angiographic RCTs, the current randomized evidence shows significantly better patency rates for RA and NT-SV compared with CON-SV, while all conduits were associated with similar rates of late mortality compared with CON-SV. NT-SV and RA were identified as the second best conduits using data from this NMA of angiographic trials.

**Table:** 

Authors’ Roles & Responsibilities	
MXD	Substantial contributions to the conception or design of the work; or the acquisition, analysis, or interpretation of data for the work; drafting the work or revising it critically for important intellectual content
HL	Substantial contributions to the conception or design of the work; or the acquisition, analysis, or interpretation of data for the work
GL	Substantial contributions to the conception or design of the work; or the acquisition, analysis, or interpretation of data for the work
MR	Substantial contributions to the conception or design of the work; or the acquisition, analysis, or interpretation of data for the work
ADF	Substantial contributions to the conception or design of the work; or the acquisition, analysis, or interpretation of data for the work
MD	Substantial contributions to the conception or design of the work; or the acquisition, analysis, or interpretation of data for the work
GDA	Substantial contributions to the conception or design of the work; or the acquisition, analysis, or interpretation of data for the work
MG	Substantial contributions to the conception or design of the work; or the acquisition, analysis, or interpretation of data for the work
SEF	Substantial contributions to the conception or design of the work; or the acquisition, analysis, or interpretation of data for the work; drafting the work or revising it critically for important intellectual content; agreement to be accountable for all aspects of the work in ensuring that questions related to the accuracy or integrity of any part of the work are appropriately investigated and resolved; final approval of the version to be published
